# An agonist of the CXCR4 receptor accelerates the recovery from the peripheral neuroparalysis induced by Taipan snake envenomation

**DOI:** 10.1371/journal.pntd.0008547

**Published:** 2020-09-08

**Authors:** Marco Stazi, Giorgia D’Este, Andrea Mattarei, Samuele Negro, Florigio Lista, Michela Rigoni, Aram Megighian, Cesare Montecucco

**Affiliations:** 1 Department of Biomedical Sciences, University of Padova, Padova, Italy; 2 Department of Pharmaceutical and Pharmacological Sciences, University of Padova, Padova, Italy; 3 Department of Medical and Veterinary Research, the Ministry of Defense, Rome, Italy; 4 CNR Institute of Neuroscience, Department of Biomedical Sciences, Padua, Italy; Universidad de Costa Rica, COSTA RICA

## Abstract

Envenomation by snakes is a major neglected human disease. Hospitalization and use of animal-derived antivenom are the primary therapeutic supports currently available. There is consensus that additional, not expensive, treatments that can be delivered even long after the snake bite are needed. We recently showed that the drug dubbed NUCC-390 shortens the time of recovery from the neuroparalysis caused by traumatic or toxic degeneration of peripheral motor neurons. These syndromes are characterized by the activation of a pro-regenerative molecular axis, consisting of the CXCR4 receptor expressed at the damaged site in neuronal axons and by the release of its ligand CXCL12α, produced by surrounding Schwann cells. This intercellular signaling axis promotes axonal growth and functional recovery from paralysis. NUCC-390 is an agonist of CXCR4 acting similarly to CXCL12α. Here, we have tested its efficacy in a murine model of neuroparalytic envenoming by a Papuan Taipan (*Oxyuranus scutellatus*) where a degeneration of the motor axon terminals caused by the presynaptic PLA2 toxin Taipoxin, contained in the venom, occurs. Using imaging of the neuromuscular junction and electrophysiological analysis, we found that NUCC-390 administration after injection of either the purified neuroparalytic Taipoxin or the whole Taipan venom, significantly accelerates the recovery from paralysis. These results indicate that NUCC-390, which is non-toxic in mice, should be considered for trials in humans to test its efficacy in accelerating the recovery from the peripheral neuroparalysis induced by Taipans. NUCC-390 should be tested as well in the envenomation by other snakes that cause neuroparalytic syndromes in humans. NUCC-390 could become an additional treatment, common to many snake envenomings, that can be delivered after the bite to reduce death by respiratory deficits and to shorten and improve functional recovery.

## Author summary

We have taken here a different angle in the search for novel treatments of snake envenomings by focusing of the phase of recovery from the peripheral neuroparalysis caused by neurotoxic snakes. This is a critical period that may require mechanical ventilation and prolonged hospitalization. We have found a drug, dubbed NUCC-390, which accelerates the functional recovery from peripheral neuroparalysis caused by the Taipan venom in mice, by acting on the CXCR4 receptor. In fact, we found here that this receptor is expressed by the motor neuron at the site of damage that, in the case of many snake venoms containing presynaptic PLA2 neurotoxins, consists in the degeneration of the axon terminal. NUCC-390 binds to CXCR4 and acts similarly to its natural agonist the chemokine CXCL12α. The compound is not toxic and can be administered after the snakebite and therefore it has the potential of becoming a non-expensive addition to the currently available antisera treatment whose efficacy is limited by the need of delivery shortly after snakebite.

## Introduction

Snakebite envenoming is a neglected disease that the World Health Organization has estimated to affect 1.8–2.7 million people each year, causing as many as 81000–138000 deaths and 400 000 cases of permanent disability, mainly in tropical and sub-tropical areas of the world [1]. In addition to death, many envenomed patients develop permanent physical and psychological sequelae, affecting their quality of life with associated increase in social costs [1, 2, 3, 4, 5]. This is aggravated by the fact that it occurs in the poor and rural parts of the world where advanced hospital care may not be rapidly available to the envenomed patient [6, 7, 8].

Major neuropathological consequences with various extent of respiratory deficits are due to envenomation by Australasian elapids, American coral and rattle snakes, Asian kraits, some cobra species and different European vipers [9–23]. In particular, Australo-Papuan Taipans cause an acute neuromuscular paralysis and impairment of the autonomic peripheral nervous system. This envenomation induces a wide range of symptoms, from respiratory and locomotor paralysis to sensory effects, that closely resemble those of botulism and of the Guillain-Barrè autoimmune syndrome. A rapid reduction of the compound muscle action potential upon electrical stimulation of motor neurons was observed in bitten patients to reach values as low as 10% of the normal figure within 30–40 hours from bite; this is usually followed by a slow recovery to 2/3 of original value within 500–700 hours [24, 25].

If respiration is not assisted, death occurs mainly by respiratory failure. Mechanically ventilated patients survive because the neurotoxins of the Taipans venom do not kill motor neurons; rather they cause degeneration of the axon terminal followed by a slow regeneration. Accordingly, envenoming may require prolonged hospitalization, but, eventually, the patient will recover more or less completely [24, 25].

In general, the peripheral neuroparalysis that arises after envenomation by neurotoxigenic snakes is due to both pre- and post-synaptic neurotoxins, with presynaptic PLA2 neurotoxins largely prevailing in determining the neuropathology caused by elapids’ envenomation, with some exception such as in the case of the Monocled Cobra (*Naja Kaouthia*) which causes an irreversible muscle paralysis via the post-synaptic blockade exerted by curarimimetic alpha-neurotoxins [26].

The Taipans snakes are highly venomous Australo-Papuan elapids which form the *Oxyuranus* genus. Among the several species of this family, the venom of *Oxyuranus scutellatus* (Coastal and Papuan Taipan) is the most studied. The main venom component responsible for the neuroparalytic syndrome is a presynaptic PLA2 toxin termed Taipoxin [27, 28]. The venoms of the other species of *Oxyranus* contain Taipoxin-like neurotoxins [29, 30, 31, 32], which consist of three homologous subunits, one of which is an active PLA2 whilst the others have lost enzymatic activity, but retain their specific binding to the presynaptic membrane thus increasing Taipoxin neurotoxicity [29, 30, 31, 32, 33]. This extensive research and knowledge qualify Taipan envenomation as a paradigm of the neurotoxic and neuroparalytic syndrome caused by the PLA2 neurotoxin containing venoms.

The PLA2s form a very large family of hydrolytic enzymes that cleave the ester bond of the fatty acid in position sn-2 site of the glycerol moiety of phospholipids [33]. The acquisition of presynaptic binding properties by mutation and selection is at the basis of the transformation of a generic PLA2 enzyme into a presynaptic neurotoxin as it concentrates its PLA2 activity on the presynapse causing loss of neurotransmission and neuroparalysis [33, 34]. In fact, the change in membrane composition and structure caused by the large production of lysophospholipids and fatty acids (LP and FA) [34] is responsible for all the biochemical and morphological changes observed at the motor axon terminals [35], including the massive discharge of synaptic vesicles not followed by vesicle retrieval and the mitochondrial swelling and rounding [36, 37]. When the concentration of LP/FA is sufficiently high, transient lipidic channels form in the presynaptic membrane allowing the passage of Ca^2+^ along its concentration gradient. Excessive cytosolic [Ca^2+^] causes mitochondrial rounding and fusion and discharge of synaptic vesicles whose proportionate retrieval is inhibited by LP/FA, as seen by electron microscopy and by *in vitro* experiments [35, 36, 37, 38]. High cytosolic [Ca^2+^] also causes the opening of the mitochondrial transition pore [38] and the activation of cytosolic hydrolases with the consequent degeneration of motor axon terminals [39]. This degeneration of axon terminals is closely similar to that caused by the pore forming neurotoxin α-latrotoxin produced by the black widow spider [40, 41, 42].

After degeneration, the regrowth of a functional motor axon terminal is rapid (about a week in young mice) and can be monitored by imaging presynaptic protein markers and by electrophysiology. This phenomenon is highly reproducible and can be used as a model to study the intercellular signaling occurring among nerve, muscle and perisynaptic Schwann cells [43]. By performing trascriptomics on neuromuscular junctions (NMJs) intoxicated by α-latrotoxin, we discovered that the degeneration of the motor axon terminal stimulates the appearance of the CXCR4 receptor on their plasma membrane tip and the expression and release of the chemokine CXCL12α by the perisynaptic Schwann cells. CXCL12α and CXCR4 form an intercellular signaling axis that promotes axonal growth and controls the rate of recovery of the physiological function of the NMJ [44]. CXCL12α has poor pharmacokinetics properties, but a CXCR4 agonist was identified by *in vitro* screening experiment*s* and dubbed NUCC-390 [45]. We found that, similarly to CXCL12α, NUCC-390 promotes the functional recovery of the NMJ, after α-latrotoxin induced axon terminal degeneration [46]. Stimulated by these findings and by the similarity of the cytosolic [Ca^2+^] induced degeneration of axon terminals caused by α-latrotoxin and Taipoxin, we decided to test the effect of NUCC-390 on the neuroparalysis induced by Taipoxin and by the Taipan venom. We found that NUCC-390 is very effective in the recovery from the neuroparalysis induced by this venom suggesting that this drug should be considered as a potential therapeutics for a faster and better regeneration of human NMJs after envenoming by Taipans, and, possibly by other snakes.

## Materials and methods

### Antibodies, reagents and toxins

The following primary antibodies were used at the indicated dilutions: anti-CXCR4 (Abcam, cat. Ab 124824, 1:400), anti-syntaxin (Synaptic System, cat. 110111.00, 1:200), anti-Neurofilament (Abcam, cat. Ab 4680, 1:800). α-bungarotoxin (α-BTx) (cat. B35451, 1:200) and secondary antibodies Alexa-conjugated (1:200) were from Life Technologies. Unless otherwise stated, all other reagents were from Sigma. NUCC-390 was synthesized as previously described [46]

Purified Taipoxin and *Oxyuranus scutellatus* total venom were from Venom Supplies (Tanunda, South Australia). We estimated that our batch of taipoxin has a mouse LD50 of about 2 μg/Kg and that the Taipan venom is about 8 μg/Kg closely similar to value reported in the literature [47].

### Ethical statement

C57BL/6 mice expressing cytosolic the Green Fluorescent Protein (GFP) under the *plp* promoter, kindly provided by Dr. W.B. Macklin (Aurora, Colorado) with the help of Dr. T. Misgeld (Munchen, Germany), were used in immunofluorescence experiments. CD1 mice weighting around 25–30 gr were employed for electrophysiological and compound muscle action potential recordings. All the experiments that involve animals were carried out in accordance with National laws and policies (D.L. n. 26, March 14, 2014), with the guidelines established by the European Community Council Directive (2010/63/EU), and were approved by the local authority veterinary services.

### Electrophysiological recordings

Electrophysiological measurements were performed in 6–8 weeks old CD1 mice anesthetized with a cocktail of xylazine (48 mg/Kg) and zoletil (16 mg/Kg) via i.p. injection [48].

### Evoked junctional potentials (EJPs)

Mice were locally injected in the hind limb with 0.2 μg/Kg Taipoxin (a dose close to the mouse lethal dose 50% which we had previously tested using the mouse diaphragm assay) with daily local injections of 3,2 mg/Kg NUCC-390 (diluted in 40 μl physiological solution containing 0.2% gelatine) or vehicle only. Seventy-two hours later mice were killed and soleus muscles were quickly excised and pinned on the bottom of a silicone elastomer coated (Sylgard 184, Down Corning) petri dish filled with oxygenated (95% 0_2_ 5% C0_2_) Krebs-Ringer solution containing 1 μM μ-Conotoxin GIIIB (Alomone, Israel), a selective blocker of skeletal muscle NaV1.4 Channels.

Electrophysiological intracellular recordings were performed at room temperature (20–22° C) on soleus muscle fibres using glass microelectrodes (1.5 mm outer diameter, 1.0 mm inner diameter, 15–20 MΩ tip resistance; GB150TF, Science Products GmbH Germany) filled with a 1:2 solution of 3 M KCl and 3 M CH_3_COOK. Signals were amplified using an intracellular bridge mode amplifier (BA-01X, NPI, Germany),

Evoked neurotransmitter release was recorded under current-clamp conditions. Resting membrane potential was clamped at −70 mV. Excitatory Junction Potentials (EJPs) were elicited by supramaximal stimulation at 0.5 Hz (stimulus duration 0.4 ms) of soleus nerve using a suction electrode (GB150TF, Science Products GmbH Germany) filled with extracellular solution and connected to a stimulator (S88, Grass, USA) via a stimulus isolation unit (SIU5, Grass, USA) in a capacitative coupling mode.

Amplified signals were digitized using a digital A/C interface (NI PCI-6221, National Instruments, USA) and then fed to a PC for both on-line visualization and off-line analysis using appropriate software (WinEDR, Strathclyde University; pClamp, Axon, USA). Stored data were analyzed off-line using the software pClamp (Axon, USA).

### Compound Motor Action Potential (CMAP)

Mice were locally injected with 0.8 μg/Kg of *O*. *scutellatus* venom (a dose close to the mouse lethal dose 50%, which we had previously tested using the mouse diaphragm assay) in the hind limb with daily local injections of 3,2 mg/Kg NUCC-390 (diluted in 40 μl physiological solution plus 0.2% gelatine) or vehicle only, and the analysis was performed after 4 and 8 days from intoxication.

Following general anesthesia, the sciatic nerve was exposed at sciatic notch and a small piece of parafilm was slid under the nerve, which was kept moist by a drop of PBS. A pair of stimulating needle electrodes (Grass, USA) were then advanced until they gently touched the exposed sciatic nerve using a mechanical micromanipulator (MM33, FST, Germany). A pair of needle electrodes for electromyography (Grass, USA) were used for electromyographic recording of gastrocnemius muscle fibres activity. The recording needle electrode was inserted halfway in the gastrocnemius muscle while the indifferent needle electrode was inserted in the distal tendon of the muscle. Compound muscle action potentials (CMAPs) were recorded following supramaximal stimulation of the sciatic nerve at 0.5 Hz (0.4 ms stimulus duration) using a stimulator (S88, Grass, USA) via a stimulus isolation unit (SIU5, Grass, USA) in a capacitative coupling mode.

Recorded signals were amplified by an extracellular amplifier (P6 Grass, USA), digitized using a digital A/C interface (National Instruments, USA) and then fed to a PC for both on-line visualization and off-line analysis using appropriate software (WinEDR, Strathclyde University; pClamp, Axon, USA). Stored data were analyzed off-line using pClamp software (Axon, USA).

### Immunohistochemistry

Anesthetized mice were locally injected with either Taipoxin or *O*. *scutellatus* venom close to soleus muscles. Muscles were dissected at different time points, fixed in 4% paraformaldehyde in PBS for 30 min at room temperature, and quenched in 0.24% NH_4_Cl PBS for 20 min. After permeabilization and 2 h saturation in blocking solution (15% goat serum, 2% BSA, 0.25% gelatine, 0.20% glycine, 0.5% Triton X-100 in PBS), samples were incubated with primary antibodies for 72 h in blocking solution at 4°C. Muscles were then washed in PBS and incubated with secondary antibodies diluted in PBS+0.5% Triton X-100. Images were collected with a Leica SP5 confocal microscope equipped with a 40× HCX PL APO NA 1.4. Laser excitation line, power intensity and emission range were chosen according to each fluorophore in different samples to minimize bleed-through.

### Statistical analysis

Sample sizes were determined by analysis based on data collected by our laboratory in published studies. We used at least N = 4 mice/group for electrophysiological analysis. We ensured blinded conduct of experiments. For imaging analysis, the quantitation was conducted by an observer who was blind to the experimental groups. Data were displayed as histograms and expressed as means ± SEM. GraphPad Prism software was used for all statistical analyses. Statistical significance was evaluated using unpaired Student’s t-test. Data were considered statistically different when *p < 0.05, **p < 0.01.

## Results

### The CXCR4 receptor is expressed following degeneration of axon terminals induced by Taipoxin

**Fig 1:**
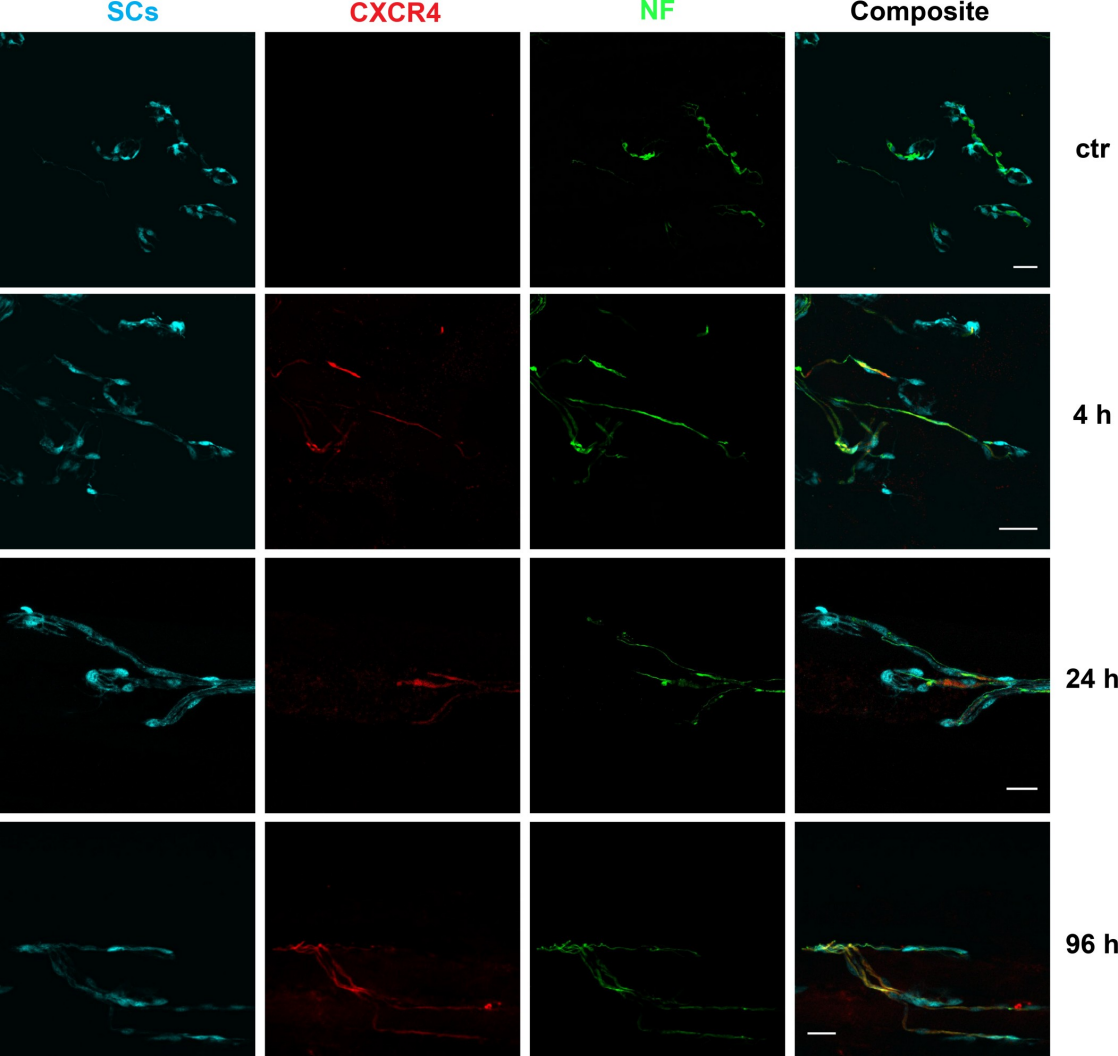
CXCR4 receptor is long expressed in mouse neuronal axons after taipoxin muscle injection. The odd rows show untreated controls while the even rows show samples of soleus muscle staining after different time periods (right) from taipoxin injections. The first column shows pictures of the perisynaptic Schwann cells expressing Green Fluorescent Protein (GFP) at the neuromuscular junction (NMJ) of the soleus muscle. The second column shows that the taipoxin injection causes CXCR4 expression (*red*) on the soleus NMJs which is increasing with time from injection. The axon terminal is identified by neurofilaments (NF) staining (third column, *green*) and neuronal degeneration with time is accompanied by a decreased staining as seen in the lower panel of the 4 h period; less so in the 24 h panel, a time period at which regeneration has already started. Scale bars: 20 μm. The same magnification was used for a line of panels and it is given in the right panel scale bars: 10 μm.

Previous studies have documented that the injection of Taipoxin, a PLA2 presynaptic neurotoxin, causes a progressive degeneration of the motor axon terminal with loss of synaptic vesicles, mitochondrial swelling and rounding and axon terminal enlargement, similar to that caused by α-latrotoxin [36, 37, 38]. This is followed by loss of the nerve terminal [40, 41] and regeneration of the NMJ by axon growth. We found that an important player in regeneration is the receptor CXCR4 [44]. Fig 1 shows that taipoxin causes a reversible nerve terminal degeneration that is almost complete within 24 hours with an ensuing substantial regrowth after 96 hours as detected by the increased extension of neurofilaments (NF, green, in the third column). The co-staining with an antibody specific for the CXCR4 receptor (red, in the second column) indicates a marked expression of this receptor by the axon stump; its level of expression progresses with time and is high even after 96 hours, a time when regeneration is robust and under way.

These data suggest that CXCR4 can be an agonist drug target to facilitate nerve regeneration after its terminal degeneration. The natural ligand of CXCR4 is the chemokine CXCL12α which acts as an axonal growth factor [44], but chemokines in general have poor pharmacokinetics due to hydrolysis or modification *in vivo*. A recent study has indicated that NUCC-390 is an agonist of CXCR4 [45] that acts *in vivo* to accelerate and improve the recovery of NMJ function after α-latrotoxin induced degeneration [46]. Therefore, we wondered whether *in vivo* administration of NUCC-390 accelerates nerve regeneration also in the case of Taipoxin intoxication.

### The CXCR4 receptor agonist NUCC-390 stimulates the recovery of function of the neuromuscular junction after degeneration of motor axon terminals induced by Taipoxin

**Fig 2:**
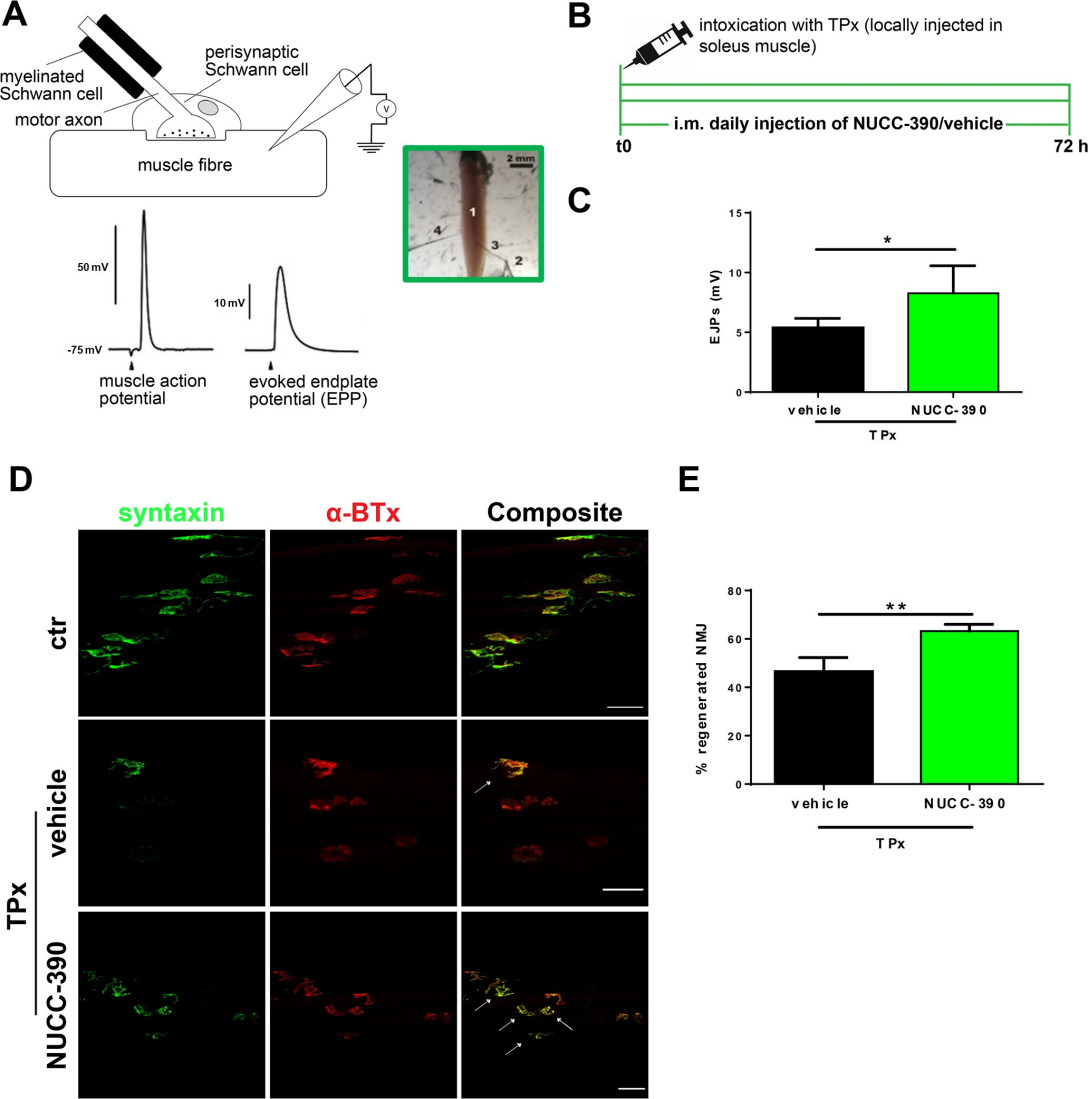
NUCC-390 promotes NMJs recovery following Taipoxin muscle injection in mice. (A) schematic representation of the technique of measurement of the Evoked Junctional Potentials (EJPs). (B) temporal scheme of the administration of taipoxin and NUCC-390 administration. (C), EJPs of soleus muscles 72 h post taipoxin injection (0.2 ug/Kg) in the hind limb, w/o daily NUCC-390 administrations. Each bar represents the mean of the EJP amplitude ± SEM from N = 5, number of analyzed fibers: 12, *p<0.05. (D), Representative immunostaining of the presynaptic marker syntaxin and (E) quantitation of regeneration of NMJs performed on the same muscles used for EJP measurements reported in C (N = 5). *p<0.05, **p<0.01. Motor axon terminal (MAT) is identified by syntaxin immunostaining (*green*) and post-synaptic AChRs by fluorescent α-BTx (*red*). Scale bars: 50 μm.

Having found that CXCR4 receptor is expressed on the axon terminal following its degeneration induced by taipoxin, we tested the effect of NUCC-390 on the damaged NMJs. We monitored nerve regeneration via electrophysiology by recording Evoked Junctional Potentials (EJPs) which allows one to estimate neurotransmitter release at single NMJ thus providing an indication of the nerve terminal functionality. Preliminary experiments showed that recovery of function after paralysis in mice is already very significant after 72 hours and this can be used as a significant time point to quantify the recovery. Fig 2 panel C shows that a daily treatment with i.m. injections of NUCC-390 (panel B) accelerates the NMJ functional recovery, In fact, after 72 hours from Taipoxin injection significantly higher amplitudes of EJPs are recorded in mice treated with NUCC-390 with respect to those injected only with Taipoxin and vehicle. The same conclusion was reached after an immunofluorescence analysis of the recovery of the expression of syntaxin which is a marker of the presynaptic membrane (panels D and E). The staining of syntaxin is rather spotty compared to the control indicating that regeneration is under way, but the positive effect of NUCC-390 is evident.

Taken together, these data strongly suggest that NUCC-390 increases the regenerative outcome on motor axons intoxicated with Taipoxin.

### NUCC-390 stimulates the recovery of function of the neuromuscular junction after degeneration of axon terminals induced by the Taipan venom

Taipoxin accounts for about 20% of the protein of the Taipan venom and it is clearly the major responsible for the neuroparalysis consequent to Taipan envenomation [31]. However, there are other components that may contribute to the paralysis of the NMJ such as Kunitz-type and three-finger toxins acting post-synaptically.

**Fig 3:**
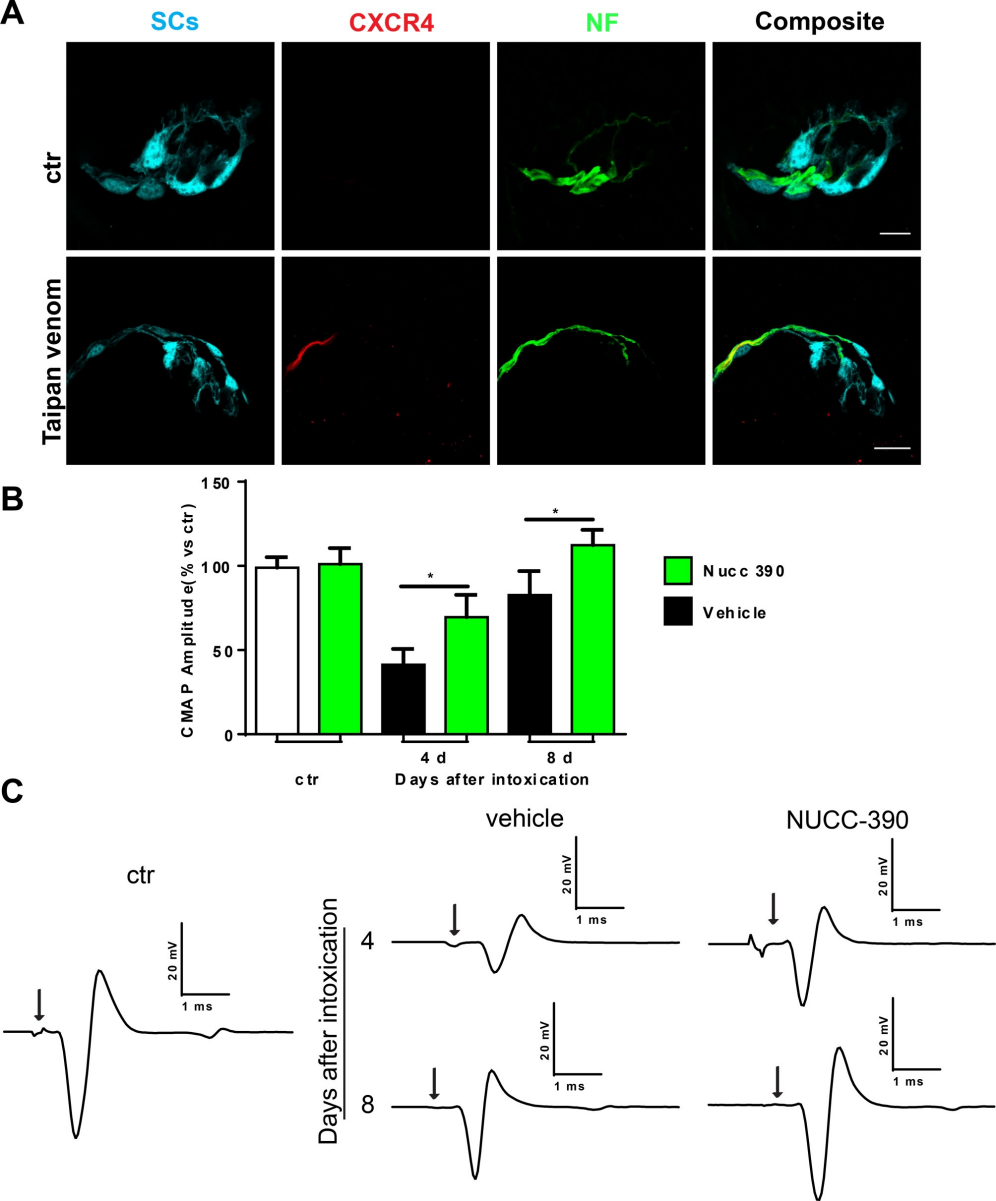
NUCC-390 promotes nerve regeneration after O. *scutellatus scutellatus* venom muscle injection in mice. (A), CXCR4 staining (*red*) at Soleus NMJs in controls and 4 h after local injection of 0.8 ug/Kg of Taipan venom. PSC are GFP-positive (*cyan*), the axon terminal is identified by NF staining (*green*). Scale bars: 10 μm. (B), CMAP values recorded on gastrocnemius muscles 4 and 8 days after injection with 0.8 ug/Kg of Taipan venom, w/o NUCC-390 daily local administration. The venom affects all the muscles around the site of the injection in mice. Data are expressed as CMAP amplitude (% vs ctr). *p<0.05. C, Representative CMAP traces of gastrocnemius muscles before and 4/8 days after hind limb intoxication (w/o NUCC-390 local treatment).

**Fig 4:**
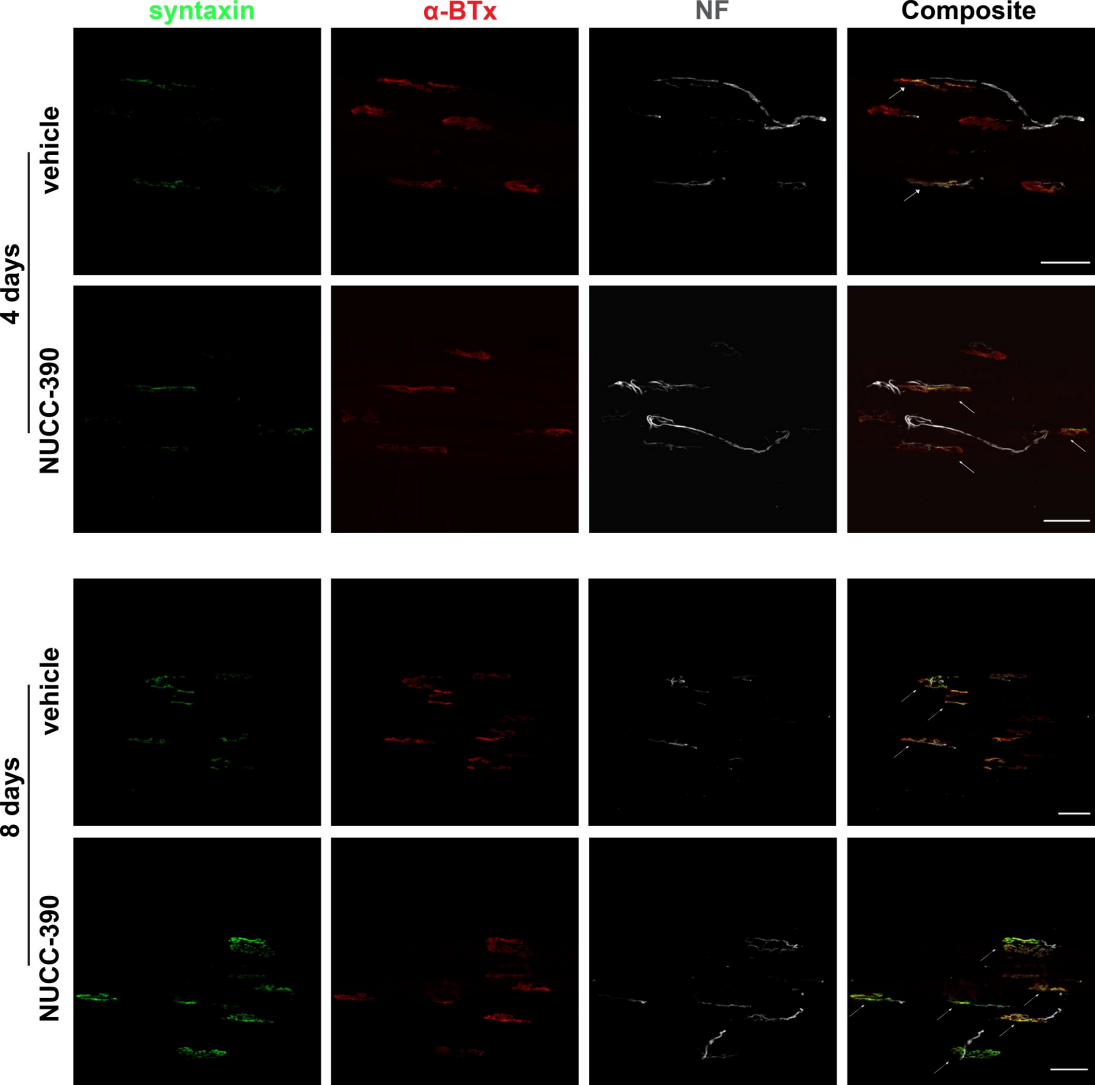
NUCC-390 promotes morphological nerve recovery after *O*. *scutellatus scutellatus* intoxication. Representative immunostaining of intoxicated NMJs after 4/8 days, performed on the same muscles used for CMAP analysis (Fig 3B and 3C). Motor neurons axon terminals are identified by syntaxin immunostaining (*green*), post-synaptic AChRs by fluorescent α-BTx (*red*) and the axon by NF staining (*white*). Scale bars: 50 μm.

Therefore, to test the potential value of NUCC-390 in promoting the functional recovery after the paralysis generated by Taipan biting, we performed electrophysiological and imaging experiments in mice hind limb muscles injected with the Taipan venom, whose results are shown in Fig 3 and in Fig 4.

As electrophysiological parameter to evaluate the recovery of physiological function, we opted for the compound muscular action potential (CMAP). CMAP was chosen because it reports quantitatively on the recovery of the function of the entire muscle rather than that of the single muscle fibre obtained by measurement of EJP. This allows one to evaluate both pre- and post-synaptic effects of intoxication. Preliminarily, we ascertained whether also the Taipan venom induces a similar expression of the CXCR4 receptor at the NMJ. Panel A in Fig 3 shows that this is the case. Accordingly, the effect of NUCC-390 was measured: panel B shows that after 4 days (about 1/3 regeneration in controls) and after 8 days (about 80% recovery in control mice) the value of recovered CMAP after paralysis is much higher in the mice treated with NUCC-390, indicating that stimulation of the neuronal receptor CXCR4 is effective in speeding up recovery after paralysis. This conclusion is further supported by an imaging analysis of the expression of the presynaptic membrane markers syntaxin and neurofilaments performed on similarly treated mice (Fig 4). Indeed, this figure shows a higher number of re-innervated NMJs in NUCC-390 treated muscles compared to untreated samples, with a higher syntaxin signal (*green*) that better overlaps with α-bungarotoxin staining of the post-synaptic muscle membrane (*red*). These data show that NUCC-390 can be considered a novel therapeutic to accelerate nerve structural and functional recovery from the neuroparalysis caused by Taipan envenomation.

## Discussion

The high number of deaths and of sequelae affecting survivors of snake envenoming has not stimulated an appropriate level of scientific interest and research aimed at finding adequate treatments. Clinically oriented research has focused on the development of antivenom antisera produced in animals, mainly horses [49, 50, 51, 52, 53]. This is a well validated therapeutic approach that, however, has some drawbacks including: i) the different protein toxin composition of the venoms produced by the many different venomous snakes, ii) the fact that in several cases the snake species responsible for the paralysis is not known. Thus the availability of a rather large range of antisera is needed [49, 50]. Additionally, this treatment is efficacious only when the anti-serum is administered soon after envenomation. Such requirement is particularly stringent in the case of venoms acting predominantly on the human nervous system [10, 11, 54, 55], given that the neurotoxin-neutralizing antibodies have to bind the toxin(s) when it is still circulating, before it binds to its acceptor or target synaptic protein. Moreover, heterologous antibodies cause adverse immune reactions in a sizeable proportion of treated patients [11]. Another classical approach is the identification of drugs able to block one or another essential aspect of the pathogenesis entrained by snake biting. This is exemplified by acetylcholinesterase inhibitors that increase the availability of acetylcholine at the intoxicated NMJ [10, 11] and by the more recent approach of using snake PLA2 inhibitors [56, 57, 58].

We propose here a novel level of intervention that focusses on the regeneration that follows the peripheral neuroparalysis caused by neurotoxic snake venoms. These venoms can act mainly pre-synaptically, or mainly post-synaptically or both at the pre- and post-synaptic levels. A prototype of the first group of venoms is provided by the Taipan snakes and contains as main neuroparalytic component Taipoxin [26, 29, 30, 31, 56, 58]. We have been driven to this study by previous findings that α-latrotoxin and Taipoxin cause a very similar degeneration of the motor axon terminal triggered by Ca^2+^ overloading of the motor axon terminal which causes, within few hours, its complete degeneration [40, 41]. Remarkably this is followed by the rapid (about a week in mice) and complete recovery of NMJ function. In particular, we have previously shown that the α-latrotoxin induced degeneration is followed by a process of regeneration during which the myelinated motor axon stump expresses higher levels of CXCR4 receptor and perisynaptic Schwann cells are activated to produce the chemokine CXCL12α. This chemokine is the natural agonist of CXCR4 and it stimulates motor axon growth [44]. More recently, we found that the recovery of function of the α-latrotoxin degenerated NMJ is also stimulated by a chemical CXCR4 agonist, dubbed NUCC-390, which is not toxic and accelerates the NMJ functional recovery similarly to CXCL12α [46].

Here we found that both Taipoxin and Taipan venom induce the degeneration of the motor axon terminal with the expression of CXCR4 at the damaged site providing the basis for testing the effect of NUCC-390. Using imaging and electrophysiological measurements, we found that NUCC-390 accelerates the functional recovery of the muscle activity. It is very likely that the present findings can be extended to envenomations by other neurotoxic snake venoms which containing as a major component a presynaptic PLA2 neurotoxin such as *Notechis scutatus*, *Micrurus fulvius* and several others [59]. This suggestion might be extended to the bites by snakes of the genus *Bungarus* which cause neuroparalysis in a very large number of cases, mainly in South-East Asia [13, 14, 15, 16, 17], and to Alpine vipers whose venom includes neurotoxic PLA2 such as ammodytoxins [60, 61]

NUCC-390 is the first drug found to be capable of speeding up the recovery of function of the neuromuscular apparatus after a neuroparalytic snake envenomation. This is particularly important in humans as recovery of the neuromuscular apparatus after Taipan envenomation requires more than a month, as judged by the measurement of CMAP [24,25]. NUCC-390 is expected to decrease the recovery time after envenomation also in humans and this would result in shortening hospital stay with a parallel decrease of healthcare costs. This is an important aspect, particularly in developing countries where most of neuroparalytic envenomings occur.
